# Learner identity in secondary post-compulsory education students from Areas in Need of Social Transformation: an example of resilience

**DOI:** 10.1007/s10212-023-00704-6

**Published:** 2023-05-19

**Authors:** R. Cubero-Pérez, M. Cubero, J. A. Matías-García, M. J. Bascón

**Affiliations:** 1 Department of Developmental and Educational Psychology, University of Seville, Seville, Spain; 2 Department of Experimental Psychology, University of Seville, Seville, Spain

**Keywords:** Adolescents, Post-compulsory education, Learner identity, Resilience, Contexts of social exclusion, School dropout intention

## Abstract

Achieving adequate integration and success at school in the post-compulsory stages involving situations where there is a risk of social exclusion is a real identity challenge for adolescents. In this research, we used a convenience sampling and selected two high schools located in Areas in Need of Social Transformation in Seville (southern Spain). We studied the learner identity of all their students in the first and second grade of secondary post-compulsory education (*N* = 70). These students present a trajectory of resilience, as they remained in the education system despite facing many difficulties. In this exploratory research, their identity as learners was analysed through an interview applied in a focus group format (*N* = 12), where their supports, strengths and psycho-social obstacles that facilitate/hinder their stay in the education system in the post-compulsory stage were also identified. Results show that adolescents have a good attitude towards academic training, based on the conviction that, in the future, they will be able to achieve a higher quality of life and a rapid insertion in skilled jobs. The image students have of themselves combines a negative perception of their lack of work habits, the difficulty of self-regulation and the little effort made, with a more positive view of their agency in the process, highlighting their intellectual and academic capacity and their effort when they set out to do so. Family, teachers and peers play a role in the resilience and identity construction of the adolescents, through protecting them, developing positive perceptions and expectations, stimulating control and effort and attributing successes and failures to students. Programmes based on the participation of the target group are essential for the design and improvement of psychosocial intervention programmes in these contexts.

In the last decade, adolescents leaving the education system have been aggravated by the great economic and social crisis experienced in Europe. In the last year, the effects of the COVID-19 pandemic, especially in the peripheral countries of the area, have also increased inequalities and the risk of social exclusion (European Parliament, 2022). In Spain, during 2021, more than 13 millions of people (27.6% of the population) were at risk of poverty and/or social exclusion (EAPN, 2021), a figure that is expected to increase considerably due to the prevailing health situation post-covid.

In order to tackle school leaving, the European Union (EU) has been working to develop and implement improvement programmes in the various education systems (European Parliament, 2022). However, the situation has not changed substantially, even more so in this last year, when the development and use of new technologies applied to education have been most necessary and, therefore, the digital divide between different population sectors has become more evident. Perhaps one of the most serious consequences is the very continuity of the students over the different levels of the education system. For more than a decade, this continuity is still considered one of the main purposes of the educational systems of Europe (Martín Quintana, et al., 2015). According to the Europe 2020 education indicators, the strategic framework for cooperation in education and training, the education quality indicators and the gender equality indicators published by Eurostat (2022a, 2022b), it is a priority objective for young people to continue their education beyond compulsory schooling. This concern is also Objective 4 of the Sustainable Development Goals: “Ensure inclusive and equitable quality education and promote lifelong learning opportunities for all” (United Nations, 2022).

In Spain, this situation poses a major problem, as the country has one of the highest rates of early school leaving (ESL) in the EU. ESL refers to students between 18 and 24 years of age who have not managed to complete post-compulsory secondary education (Fernández, et al., 2010). In 2022, the dropout rate in Spain was as high as 16.7% for boys and 9.7% for girls, whereas in Europe, these percentages were of 11.4% and 7.9% respectively (Eurostats, 2022a). These statistics increase in the case of Andalusia, where the present study is carried out. Specifically, the dropout rate in 2022 for males in Andalusia was 18.4%, and for females, it was 12% (Eurostats, 2022b). These are therefore students who have not enrolled in a Baccalaureate programme or in intermediate level training courses or who have dropped out without completing these courses. To talk about school leaving is to talk about the interruption of the social trajectory of young people who, after completing compulsory secondary education and without any prospect of continuing their studies, are deprived of being able to develop the essential skills required by the knowledge society (Vélaz de Medrano & De Paz, 2010).

Tackling this problem involves the joint and coordinated intervention on many factors in the students’ lives. Among the environments considered “disadvantaged”, we find the Areas in Need of Social Transformation (ZNTS) (Junta de Andalucía, 2022). These places are defined as specific, physically delimited urban spaces where the population lives in structural situations of serious poverty and social marginalisation and where there are significant problems in areas such as infrastructure and public services, school absenteeism and failure, unemployment and lack of training and health and hygiene deficiencies. In these contexts, much research has been carried out on ESL so far, although little of it focuses on the perspective of its main actors: students (Ferguson, et al., 2005; Fernández et al, 2010; Rumberger, 2001). This is especially important in the ZNTS, where the percentage of students in post-compulsory courses is quite low (Cuberos et al, 2021; Junta de Andalucía, 2022; Rosales-Migliore & Cubero-Pérez, 2021).

Consequently, achieving an adequate integration and school success in this complex system is a real identity challenge for these adolescents (Rosales-Migliore & Cubero-Pérez, 2021). At present, there is limited research about resilient students’ identity as learners and the protection factors that allow such adolescents to continue attending the educational system. We elaborate on these two ideas that are the focus of our investigation below.

Although the selves of students develop in a multiple and changing set of identities (Yuste, et al., 2021), the learner identity is the most relevant for our study (Coll & Falsafi, 2010; Rosales, 2015; Wenger, 2000). Learner identity is shaped by the understandings people have of themselves (Burke, 2006) and the understandings others have of them as learners. These are the perceptions that will influence future achievements and failures (Engel & Coll, 2021). In Lawson’s terms (2014), it is defined as “how an individual feels about himself/herself as a learner and the extent to which he/she describes himself/herself as a ‘learner’” (p. 344).

Learner identity arises from the relationships established with other people in learning contexts, so it is constantly being reconstructed (Engel & Coll, 2021). The social dimension is essential in the construction of identity, as well as the connection between the understanding as a learner and the context where the activity takes place, in which a group feeling of belonging and recognition is built (Bernstein & Solomon, 1999). Analysing this identity involves knowing the process by which people come to make sense of their participation in learning activities by recognising themselves as learners, as well as the values and emotions that accompany them (Gee, 2000).

Despite the identity challenge posed by development in risk contexts with high conflict and very poor resources, in these environments, it is possible to find students who can be defined as a resilient population. Resiliency can be defined as the presence of protective factors against adverse circumstances and risk (Luthar et al., 2000). By facing this challenge, young people develop specific skills in the construction of their identities to cope with inequalities and social exclusion, using a set of resources that can be emotional, social and cognitive, which allow them to remain in the education system (Olsson et al., 2003). The presence of such protective factors also determines the emergence of other future protective factors for the individual (Tiet et al., 2010; Wenger, 2000). In the longitudinal study by Tiet et al. (2010), adolescents were considered resilient if they fit into contexts where there are elements of risk, while presenting good academic work, adequate psychosocial functioning, high self-esteem and the absence of antisocial behaviour. Therefore, in the educational field, resilience refers to the adaptive psychological, emotional and behavioural responses that students have in spite of the adversity of the context surrounding them, which allow them to achieve academic success Then, resilience does not eliminate risk factors but allow the individuals to effectively deal with them (Wenger, 2000). We consider students to be resilient to the extent that they present a resilience trajectory having remained in the educational system beyond compulsory education in risk contexts. This definition is consistent with that of authors such as Lessard et al. (2014), Longás et al. (2019) and Masten (2007).

It is essential to highlight that resilience must be understood as an interactive and dynamic process that involves individuals, families, schools and communities (Gartland et al., 2019; Toland & Carrigan, 2011). It is not a capacity or a characteristic of the individual taken apart from setting and, of course, it does not make those people who present it in a certain context under certain conditions invulnerable. If the conditions change, the difficulties increase and/or the context changes, individuals who show resilience may stop showing it. In this sense, the concept of resilience constitutes a definition of the situations of positive adaptation of the individual in the face of adversity, and not an explanation of said adaptation (Matías-García, 2022). Thus, resilience can be conceptualized as the product of the interaction between risk and protective factors (Olsson et al., 2003). The promotion of students’ resiliency is then an important goal of school mental health practices (Szlyk, 2020).

We understand that the study of resilient students must be approached from a preventive and positive model, whose objective is to identify factors that promote and protect students (Benson et al., 2006; Rodrigo et al., 2015). The challenge for the institutions is to contribute to the healthy construction of a learner identity that allows students to be aware of their performance as learners and to face new learning by optimising their possibilities, reducing their difficulties and taking their previous experiences as a reference. As some authors state (Rosales-Migliore & Cubero-Pérez, 2021), if learning experiences and activities are positive and help to form a healthy learner identity, young people will be more likely to succeed in the education system. Rosales and Cubero (2016) define the identity of a healthy learner as one which includes certain indicators. These indicators are, among others, the students’ awareness of their role and performance as a subject of learning, the ability to narrate their experience as a learner in a positive and reflective way by incorporating positive experiences, the ability to engage in their learning process as part of a larger group, the ability to find meaning in the learning activity to be carried out and the perception of themselves as unique persons who relate positively to others and perceive others as significant and important for their own learning.

This study aims at investigating learner identity and resilient students in risk contexts. Our research questions are (1) what is the learner identity expressed by groups of students who present a trajectory of resilience in ZNTS (Areas in Need of Social Transformation) and (2) what are the psycho-social strengths and obstacles that groups of students identify, which facilitate/hinder their retention in the education system, given the current lack of research of this nature. This involves analysing how students deal with identity challenges according to their own perception and experience. The specific objectives of this work are as follows:

1. To describe the reasons that these students identify as responsible for them remaining in the education system.
2. To describe the supports that students perceive they have had in order to remain in the education system.
3. To identify how these students describe themselves as students.
4. To identify the strengths that these students perceive themselves to have as learners.
5. To identify the obstacles and difficulties that students perceive they have to continue to be enrolled within the education system and how they have overcome them.
6. To analyse the perception that students believe their family, friends, teachers and the neighbourhood have of them as students.
7. To describe the future projection of the students.

## Method

### Participants

We sampled the schools located in Areas in Need of Social Transformation with poor performance in Seville (Southern Spain). We used convenience sampling and selected two of them, who agreed to participate in the study.

The difficulties and challenges these schools’ students face make them significantly more vulnerable to school failure and early school leaving. According to the data provided by the Spanish National Institute of Statistics, the population living in their neighbourhoods consists of 18.8% of children aged 0 to 14, 67.12% of people aged 15 to 65, and 14.08% of people over 65. In terms of employment and economic level, there is a high percentage of unemployment, with most families being dependent on street selling and businesses operating in the informal economy. Regarding the educational level in both neighbourhoods, there is a high rate of school failure and absenteeism for both genders.

In our study, we focused on the students from these areas that stay within the education system. Thus, we took all the first- and second-year students of post-compulsory education of the two aforementioned schools. Following Masten’s (2007) definition, we considered these students present a trajectory of resilience because they completed compulsory education and continue in the education system beyond that level, showing proper academic performance in the face of risk. Other studies have adopted the same definition of students with a trajectory of resilience (Lessard et al., 2014; Longás et al., 2019).

A total of 70 students were studied, with an average age of 17.4 (SD = 1.45). In total, 57.1% students identified themselves as male and 42.8% as female. A total of 45.7% participants belonged to the first year of Post-Compulsory Secondary Education and 54.2% to the second year.

The educational level of the parents is predominantly low, with 29.9% with no studies of any kind, 28.7% with basic studies (primary and/or secondary), 20.7% with intermediate studies (post-compulsory secondary education or intermediate vocational training) and 20.7% with higher studies (higher education or university studies). Of these people, an average of 1.33 people have an income of some kind. In 16% of the participants’ families, no one contributes to the economic income. In families where there is income, it often comes from low-skilled or temporary jobs. In total, 42.2% have jobs with low or no qualifications and/or little stability, 29.4% have jobs with medium qualifications or that are obtained on the basis of merit or through civil service examinations and only 2% have jobs with high qualifications. A total of 25.5% are unemployed or housewives, and 1% are retired or have a pension.

Twelve mixed-gender focus groups were formed for data collection, taking into account the situation in each school. The groups were made up of between 4 and 8 participants, with an average of 3.33 male students (SD = 1.56) and an average of 2.5 female students (SD = 1.09). The groups were formed by students from the same classroom.

### Data collection tool and technique

Data were collected through a semi-structured interview called “LIRESI” (Learner Identity Resilient Students Interview), designed for the occasion by the research team. The interview features 16 questions, divided into 2 dimensions. To answer the specific objectives 1, 2 and 5, the first dimension aims to explore the reasons why students are and remain in the education system, as well as supports and difficulties that they have encountered in their trajectory. To answer the specific objectives 3, 4, 6 and 7, the second dimension explores their identity as learners, that is, it explores their perceptions of themselves as learners, their future projections, as well as their perceptions of the views of all those members of the community who are relevant to their education in one way or another (their parents, teachers, peers and people in the neighbourhood).

During the development of the interview, both dimensions included questions related to the search for specific narratives of their experiences. They were asked several times for real examples and anecdotes that happened to them related to the topics analysed during the interview.

The interview was carried out through focus groups. The focus group is a technique used to obtain qualitative data from groups of participants through a semi-structured group interview and the dialogue between the researcher and the participants (Barbour, 2013; Beck et al., 2004; Kitzinger, 1994, 1995; Stewart & Shamdasani, 2014). According to some authors (Díaz-Dorner, 2013; Rosales, 2015), this methodology is particularly suitable for the study of students from a context in need of social transformation. As their experiences stray from the norm, they may find it more difficult to talk about their own using other methodologies, such as individual interviews (Cuberos et al., 2021; Díaz-Dorner, 2013; Rosales, 2015).

This methodology offers several advantages. The open and flexible design of the technique aims to facilitate for the researcher to explore in depth the knowledge and experience of the participants. It creates an atmosphere that can provoke enriching discussion and reflection and cause active participation. Encouraging the exchange of shared stories among participants also promotes group cohesion and richness of content (Kitzinger, 1994; Stewart & Shamdasani, 2014). In these situations, the ideas and views of each participant are spontaneously and honestly presented and discussed, alongside arguments and counter-arguments based on their experiences (Barbour, 2013; Hamui-Sutton & Varela-Ruíz, 2013; Stewart & Shamdasani, 2014).

### Procedure

Prior to the completion of the interviews, a pilot study was carried out to check whether the interview, applied in the form of a focus group, would generate the type of information we were looking for with the depth needed. Some modifications were made to facilitate its application. For instance, we simplified and reduced the number of questions to accommodate for the time limit set by the schools. In addition, some questions, specifically those related to gender differences, were seen as redundant by the participants, so we merged some of them to facilitate its answering without losing depth.

The application of the interviews in the two schools was carried out by members of the Human Activity Laboratory, who were jointly trained in how to conduct the interviews. All the focus groups of a class were conducted in one visit. When the researchers arrived at the school, they formed mixed-gender real groups of students who knew each other (groups that had a shared history prior to the research period). Real groups facilitate participation and fluid communication by sharing stories and previous experiences together (Kitzinger, 1994; Stewart & Shamdasani, 2014). Each moderator led his or her group to a different room within the same school, where the focus group was conducted. The participants were seated in a circle, with the moderator as one of the circle, in order to avoid the formation of subgroups and dominant members (Stewart & Shamdasani, 2014).

The moderator raised the issue to be explored by asking a question. The students could respond in no particular order and participate freely in interaction with the moderator and peers. The moderator was also responsible for the elicitation of answers from those students with less participation, the positive verbal reinforcement of the verbal productions, the asking of clarification questions, the synthesis of different participation turns and the signaling of contrary points of view to facilitate the interaction between participants (Stewart & Shamdasani, 2014) . Each focus group interview took between 40 and 70 min. The interviews were audiotaped and transcribed in their entirety according to the notation system proposed by Cubero et al (2008), adapted from Jefferson’s system (Atkinson & Heritage, 1984; Sacks et al., 1974). In order to protect the anonymity of the participants, different pseudonyms for students and high schools were introduced during the transcription process.

### Data analysis

A category system based on the interview transcripts was developed to conduct a content analysis. The categories were constructed a posteriori, based on the participants’ statements. This was achieved by applying techniques such as the constant comparative method (coding and simultaneous analysis) and by establishing relationships between observed categories through a process of iterative analysis until saturation, as employed in Grounded Theory (Clarke, 2005). In this way, we analysed the identity of the students attending to how they construct it during discourse.

The statements were segmented and analysed using the unit of meaning as the unit of analysis. A unit of meaning is a unit of discourse that contains an idea, which may or may not coincide with the syntactic unit of the present statement or statements (Elliott & Timulak, 2005). A new unit of meaning begins when there is a change in the theme of the expressed content. A sentence may contain one or more units of meaning.

The category system was built up through several phases of category creation, system implementation, inclusion of new categories and revision, improved definition and reorganisation of old definitions, until adequate reliability and system development were achieved. Four researchers were involved in coding the category system. Four focus groups (33% of the sample) were independently coded by these researchers to calculate the kappa coefficient as an indicator of inter-judge reliability. This coefficient indicates whether the coding system was systematically applied in the same way by the four researchers, correcting for random agreements. Values between 0.86 and 0.93 were obtained, which are considered very good value within the scientific literature (Cohen, 1960).

The final category system presents 64 categories organised into 15 different dimensions, which are related to the questions in the conducted interview. These dimensions offered information on one or more specific objectives of our research questions. These dimensions were the following:

1. Why you are here. Reasons to be in the education system (specific objective 1).
2. Support and help (specific objective 2).
3. Perception of yourself as a student (specific objective 3).
4. Strengths you identify as a student (specific objectives 3 y 4).
5. Weaknesses and difficulties encountered (specific objective 5).
6. Strategies for overcoming difficulties (specific objective 5).
7. Parents’ perception of you as a student, according to your view (specific objective 6).
8. Teachers’ perception of you as a student, according to your view (specific objective 6).
9. Peers’ perception of you as a student, according to your view (specific objective 6).
10. Neighbourhood people’s perception of you, according to your view (specific objective 6).
11. Emotions in the face of what people say about your neighbourhood (specific objective 6).
12. Perception of your neighbourhood (specific objectives 2, 5 and 6).
13. Coping strategies for negative neighbourhood discourses (specific objectives 4, 5 and 6).
14. Future perspective (specific objective 7).
15. Alternatives to secondary post-compulsory education (specific objective 7).

In this way, it was possible to collect relevant information about the participants’ educational trajectory, referring to the past, present and future of their role as students. Likewise, we obtained information about their own self-perception as students, as well as information related to what they manifest as perceptions of the significant others about them.

## Results

In the following section, we will discuss the results for each dimension analysed. The data will be expressed in terms of percentage, using two indicators. The first indicator is the percentage of categories within each dimension, as an indicator of the extent of appearance of that category in the set of interviews. The second indicator is the percentage of focus groups in which that category has been coded at least once. This will serve to check the dispersion of the category across the focus groups, so that we can see whether it has appeared widely or only in some groups. The most relevant categories within each dimension will be discussed.

In the analysis of the first dimension, we can see *the reasons for continuing post-compulsory studies* (Table 1).

**Table 1:** Dimension 1 percentages: why you are here. Reasons to be in the education system

Categories	Percentage of categories	Percentage of focus groups
1.1. Quality of life and a better future	24.28	83.3
1.2. Preliminary step in a plan	18.7	58.3
1.3. My parents	16.8	58.3
1.4. Training	10.3	50
1.5. Personal growth and overcoming stereotypes	10.28	41.7
1.6. Job satisfaction and positive work expectations	6.54	25
1.7. Avoiding idleness	5.61	25
1.8. Restructuring of a personal study plan	5.61	8.33
1.9. Because of my teachers	1.87	16.7

The second column refers to the percentage of focus groups where the categories arose

These students clearly express their future goals, and the need to pursue a better life, for which they place education as vital to their achievement. In a large number of cases, parents have supported or encouraged them to study and continue. Personal growth, as well as the overcoming of stereotypes—such as those referring to the Roma ethnic group—are other reasons that make them stay in the education system. These categories were highly visible both in terms of the extent of discussion and the distribution of the different focus groups (Table 1).

As for the second dimension, *support and help*, the participants mentioned different agents (Table 2).

**Table 2:** Dimension 2 percentages: support and help

Categories	Percentage of categories	Percentage of focus groups
2.1. My parents	28.86	100
2.2. Teachers, principal, school	21.4	83.3
2.3. My peers, my colleagues	19.3	75
2.4. My family	12.83	83.3
2.5. I am my own support	10.2	58.3
2.6. Financial support	3.74	33.3
2.7. My partner	2.67	33.3
2.8. I have no support	1.07	8.3

The second column refers to the percentage of focus groups where the categories arose

Parents, family, teachers, classmates and friends serve as emotional support and clearly insist that they must continue their studies. They also mentioned that one of the most important sources of support is themselves, as they are focused on their objectives and learn from their own experiences. These statements were common to most of the focus groups and were discussed in depth, with students mentioning numerous personal stories about these supports. It is interesting to note that they rarely mentioned a lack of any support.

In the third dimension, we analysed the discourse related to the *participants’ perception of themselves as students*. Most self-descriptions were negative (71.2%). This negative perception was shaped in terms of effort: they were disorganised, lazy, distracted or disinterested. In terms of positive perceptions of themselves (28.8%), they mainly mentioned that they had good skills, were good students and were proud of themselves. Although most groups commented on both positive (75%) and negative (83.3%) elements, negative elements were more common.

The fourth dimension complements the previous one, gathering in a more specific way all those *strengths that the students identified within themselves* (Table 3).

**Table 3:** Dimension 4 percentages: strengths they identify as students

Categories	Percentage of categories	Percentage of focus groups
4.1. Habits	63.6	58.3
4.2. Ability to memorise	12.1	33.3
4.3. Ability to pass	12.1	25
4.4. Maturity, capacity for effort, feeling of responsibility	9.09	8.33
4.5. Good interaction with teachers	3.03	8.33

The second column refers to the percentage of focus groups where the categories arose

The most common strength mentioned was the study habits that a good number of them have. They also mentioned their ability to memorise and to pass, and, to a lesser extent, maturity, capacity for effort and responsibility. These statements were also the most common in most of the focus groups.

The fifth dimension identifies the *weaknesses and difficulties that students face in their educational trajectory* (Table 4).

**Table 4:** Dimension 5 percentages: difficulties encountered

Categories	Percentage of categories	Percentage of focus groups
5.1. Characteristics of the education system	21.9	41.7
5.2. Lack of study habits	21.2	66.7
5.3. Lack of learning strategies	16.8	66.7
5.4. Toxic relationships, bad company	9.49	41.7
5.5. Financial difficulties	9.49	33.3
5.6. Personal disinterest	6.57	41.7
5.7. Self-imposed standards	5.11	41.7
5.8. Immaturity	4.38	33.3
5.9. Traumatic situations and loss of personal support	2.92	16.7
5.10. Negative assessment about the students’ abilities made by the context	2.19	8.33

The second column refers to the percentage of focus groups where the categories arose

The three most common difficulties were the very characteristics of the education system, the lack of study habits and the lack of a learning strategies. Groups of students also often called themselves lazy and slackers. Other difficulties discussed were the financial difficulties. Their families often struggle for money and studying often means that they cannot contribute to the family economy. Sometimes, bad relationships hindered their academic development, like the presence of bad friends, bad partners or a bad relationship with their parent’s partner. These statements were also the most common in most of the focus groups. However, there were issues in these groups that, although not discussed very often, did appear in more than a third of the groups analysed as difficulties in remaining and progressing successfully in the school system: personal disinterest, self-imposed standards, and immaturity.

In the sixth dimension, the *strategies for overcoming these difficulties* were analysed (Table 5).

**Table 5:** Dimension 6 percentages: strategies for overcoming difficulties

Categories	Percentage of categories	Percentage of focus groups
6.1. Improvement of habits	74.1	75
6.3. To prove personal abilities in the face of criticism	13.8	50
6.4. External school reinforcement	6.9	16.7
6.2. Having the support of the teaching staff	5.17	8.33

The second column refers to the percentage of focus groups where the categories arose

Their main strategy was personal habit improvement, which was discussed to a greater extent and in more focus groups. This improvement in habits included better time management, organisation, attendance, completion of schoolwork, increasing study time in the subjects they have the most difficulty with and controlling those things that distract them. Half of the groups also frequently mentioned a desire to prove their own personal abilities in the face of criticism and difficulties.

In the seventh dimension, we analysed the *perception that the students’ parents* had about them, according to students’ statements. Both negative (44.3% of the utterances) and positive (55.7%) perceptions were often described by the participants, with both perceptions appearing in 83.3% of the groups analysed. In families where this perception was negative, the participants mentioned that their parents thought they were lazy or bad at studying. On some occasions, there were even insults or derogatory remarks towards their children. However, many other parents had a positive view of them as students, describing them as hard-working students and expressing pride in them.

The eighth dimension of analysis is that which refers to the *teachers’ perception of them as students, according to students’ accounts*. On the one hand, they mentioned that teachers had a negative notion of them as students (54.7%), considering them lazy, immature or childish, sometimes referring to poor attendance or bad behaviour in class. But, on the other hand, teachers had a positive perception of them as people (45.3%). The participants stated that the teachers recognised the merit in wanting to continue with their studies. These themes emerged in 83.3% and 75% of the groups, respectively.

The ninth dimension analysed their *classmates’ perception of them as students,* according to students’ views. Here, there was a stronger positive perception of their colleagues (63.3%) rather than a negative one (36.7%). Frequently, the participants mentioned that they support each other, encouraging each other to study and continue when there were greater difficulties. The negative perception was mostly in terms of effort, of being lazy and slackers. These issues were addressed in at least 50% of the focus groups analysed.

The tenth dimension referred to what students declared as the *perception that the people of the neighbourhood have about themselves*. In 43.8% of the discourses, the participants said that the people in the neighbourhood had a negative perception of the benefits of studying in everyday life. However, in 56.3% of their utterances, the students spoke of how they perceived the admiration of the people of their neighbourhoods just because they keep going to school. Both statements were common to most of the focus groups (positive: 66.7% of the groups, negative: 58.3% of the groups).

The eleventh dimension recorded the *feelings and emotions aroused in them by negative discourses about the people of their neighbourhood*. The most common emotions were negative (78.3%), expressing rejection, discomfort or disagreement. Indifference to this perceived stigma was sometimes mentioned (21.7%). However, both types of feelings appeared in only 25% of the focus groups analysed.

The twelfth dimension relates to *their own perception of their neighbourhoods*. This perception was mostly negative (81.8%), mentioning difficulties in coexistence, fights, drugs, and other negative circumstances. However, in certain cases, the participants spoke positively of their neighbourhood (18.2%), claiming that, despite its bad reputation, there were good people who lead normal lives. This issue was addressed only by a minority of the groups (16.7% and 8.33% respectively).

The thirteenth dimension gathered all those *coping strategies in the face of negative neighbourhood discourses* (Table 6).

**Table 6:** Dimension 13 percentages: coping strategies against negative neighbourhood discourses

Categories	Percentage of categories	Percentage of focus groups
13.1. Questioning/defying the opinion of others	31.6	25
13.2. Remaining silent	21.1	25
13.3. Ignoring	21.1	25
13.4. Getting angry; verbal abuse	15.8	16.7
13.5. Physical violence	5.26	8.33
13.6. Giving explanations to others	5.26	8.33

The second column refers to the percentage of focus groups where the categories arose

The most frequent were the questioning of those negative opinions and arguments against the negative discourses of people from the neighbourhood, remaining silent or simply ignoring those discourses. There were also discourses expressing great verbal abuse towards such neighbourhood opinions, although they were less frequent. Such themes appeared in a minority of the focus groups analysed.

The fourteenth dimension recorded their *perspective about their future*. All the focus groups mentioned university degrees, higher or intermediate degrees, and civil service examinations, which they expected to take in the future, representing 78.3% of the utterances in this dimension. More than 50% of the groups talked about how many participants had not yet decided what they were going to do, while in 25% of the groups, some mentioned other professions not related to further studies.

The fifteenth dimension included the different *alternative plans to the formal secondary school education* (Table 7).

**Table 7:** Dimension 15 percentages: alternatives to secondary school

Categories	Percentage of categories	Percentage of focus groups
15.1. Working	46.7	58.3
15.2. Developing an alternative within the education system	26.7	41.7
15.3. Trying again	15.6	25
15.4. Going to another country	8.89	8.33
15.5. Does not know	2.22	8.33

The second column refers to the percentage of focus groups where the categories arose

The most common plans were those aimed at finding a job, usually related to their parents’ jobs, or at finding an alternative within the education system, such as trying to complete an intermediate vocational training programme or less demanding degrees. A smaller percentage stated that if they failed, they planned to try again until they passed. These three categories appeared in more groups, especially the first one.

## Discussion and conclusions

In this research, we studied the learner identity of groups of secondary post-compulsory students from two schools located in Areas in Need of Social Transformation. These students, despite facing a multitude of difficulties related to higher school failure and early leaving, resiliently remained in the education system. Their learner identity has been explored through an interview applied in a focus group format, both addressing their trajectory through the education system and their future objectives. The data and results obtained come from a content analysis of the activity of the students in those focus groups and have been expressed in terms of the categories identified in the descriptions and narratives of the participants.

Regarding the first of the two fundamental objectives of this research, that is, what is the learner identity of students who present a trajectory of resilience, the categories identified in the conversations of the student groups show an attributional pattern (Weiner, 1985) based on personal effort, feeling of responsibility for their own actions, desire for academic success and high expectations (Burke, 2006) about their academic and professional future. They attribute their failed academic results to a lack of effort, to a bad management of their time and their will, while they consider themselves agents of their achievements, revealing internal control.

The image that students have of themselves as learners—which we define as a learner identity, following Coll and Falsafi (2010) in terms of the understanding that people have of themselves (Gee, 2000)—is both positive and negative when it comes to characterising their abilities and their academic school behaviour. When defined in negative terms, the students point to the lack of organisation in the development of tasks, excessive distractions and poor time management, increased by their lack of interest in some subjects. Their main obstacles are the lack of work habits and routines. As strengths and resources in coping with such difficulties, participants cite changing habits and routines to achieve their goals, as well as personal responsibility and effort. They consider themselves capable in academic and intellectual terms, never stating that their difficulties are due to a lack of ability. In fact, when defined in positive terms, they emphasise the importance of such work habits. They focus their discourse on their efforts and self-regulatory capacities, which are an essential pillar in their academic success (Burnette et al., 2013; Sarrasin et al., 2018; Sisk et al., 2018; Yeager & Dweck, 2012).

The fact that they attribute their failures to lack of effort and self-regulation, while they perceive their success in terms of abilities and effort (Weiner, 1985), is a clear indicator of educational resilience. As research on development throughout the life span has shown, the perception of control over one's life in terms of causal attribution, locus of control and perceived competence contributes to overcoming difficulties (Skinner & Zimmer-Gembeck, 2010). Studies also show that interventions on students’ beliefs trying to favour a conception that effort is capable of developing and improving intelligence, are the ones that enhance resilience, improve performance and decrease the probability of failure and dropout. This is especially true of students who are at high risk of dropping out of the education system (Sarrasin et al., 2018; Sisk et al., 2018; Yeager & Dweck, 2012).

The results obtained allow us to state that the groups of adolescents studied have a good attitude towards education and academic training, and that this attitude is based on the conviction that, in the future, they will be able to have a better quality of life, and, thanks to their studies, they will be able to opt for rapid insertion in qualified jobs. In fact, the perception of these students in a context of social exclusion—with parents who are unemployed or working in precarious jobs—coincides with the data we have on school failure and employment. Marchesi (2003) indicates that people who have failed at school have greater difficulty in finding stable jobs with optimal salaries. This factor is shown to be a clear determinant in the expectations of the participants in our study.

In terms of future projection, the students in our research show a great orientation towards defined goals, since in every focus group, students extensively describe plans for the future. Participants express their determination to continue studying, some of them going on to university. In addition, they define the work and family situations that they intend to achieve, so current personal choices are part of a plan and objectives they are pursuing. In this way, they find meaning and future in the education they receive, which are necessary characteristics in the development of a healthy learner identity (Rosales & Cubero, 2016). We can state that the characterisation of the identity of adolescents in risk contexts who remain in the non-compulsory education system shows a profile defined by high expectations about their future and their goals (as also confirmed by data from Burke, 2006), as well as an interest in persevering in their efforts to achieve an affluent life with a good salary. It is this expectation that gives the most strength to the planning of the present moment and to the resilience in the education system within a context dominated by the very opposite profile. For the participants, these goals are achieved through more effort and more organisation on their part. In none of the situations discussed in the focus groups do profiles showing a lack of intelligence or cognitive ability appear; in most cases, if not all, students recognise themselves as being able to control the achievement of goals with actions that are dependent on them, and they depend on them because what the students need is to make more effort and to regulate themselves better.

Concerning the second main objective of this research on the psycho-social strengths and obstacles that facilitate/hinder permanence in the education system, students’ discourses about the role of their effort and work are built on interaction with the very agents that have supported their educational continuity: family, teachers and peers (Cuberos et al., 2021; Gunderson et al., 2013; Matías-García, 2022; Moorman & Pomerantz, 2010; Park et al., 2016). In view of the aid they have received throughout their educational training, the role of the family, teachers and peers as trusted persons who advise and guide the teaching–learning process is highlighted. These various agents protect and encourage students to continue studying, which is consistent with the literature (Bianchi et al., 2021). In addition to socio-economic level, ESL is associated with educational supervision of parents, their unemployment status, family atmosphere and family communication (Martín Quintana et al., 2015). Likewise, the participants in our study shed light on the coherence between school culture and their family culture, fostering an emotional bond, the development of their learning identity and educational continuity. The role of teachers (their closeness, their empathic capacity, their authority and the motivation and guidance they provide) is also vital for adolescents (López & Urraco, 2017; Szlyk, 2020; Tarabini & Curran, 2015a, 2015b). Primary developmental contexts, therefore, constitute fundamental microsystems for school failure and/or success, since their intervention affects personal expectations and school performance (Burk & Laursen, 2005; Lozano, 2003). On the other hand, the crucial role of the immediate context is also shown in the difficulties that a non-stimulating environment poses for staying in the education system. The participants in our study say that the demotivating characteristics of the education system and the toxic relationship with people in their environment, such as classmates and siblings, have caused them difficulties in their studies.

Participants state that their families describe them in a positive light. Mothers and fathers are proud of their children for fighting for their future and not leaving the education system, although they do not forget to remind the students that they must be more diligent and insist that they strive towards and pursue their goals. They support their children in continuing their education, mainly by using a discourse associated with effort and by valuing and giving importance to school. Teachers show a negative perception of them as learners according to their dispositions not their capacities, deeming them lazy and unmotivated. In spite of this, they are support elements that, in some personal stories, have been and are determining factors for the permanence in the education system. Teachers encourage, interpret failure as a lack of dedication and effort and offer material support and personal and individualised commitment. On the other hand, participants state that their peers admire them because they are able to continue their studies despite the family problems and other adverse situations they face in their situation. Finally, the neighbourhood dimension shows a dual perspective towards the students. In certain cases, schooling involves too much effort for the benefits it presents. In others, they show a good attitude towards the students, with the school being seen as a way out of their precarious situation. The students internalize those semiotic instruments found in the social discourses of all these close agents (Santamaría & Martínez, 2005) , which influence the development of their identity as learners and their academic performance (Burk & Laursen, 2005; Dweck, 2000; Gunderson et al., 2013; Lozano, 2003; Matías-García, 2022).

Looking at this last aspect in more detail, students in the Areas in Need of Social Transformation also face certain stigmatising discourses coming from inhabitants of other neighbourhoods. Although they are aware of the difficulties that their own neighbourhoods present, these unfavourable discourses awaken negative emotions in the students, who do not see themselves in that light. Sometimes, they have to face negative assessments of their abilities or certain stereotypes associated with them, such as those related to the Roma community. According to the literature, how students cope with the discourse of failure determine their continuity in the education system (Yeager & Dweck, 2012). Our data show that the strategies and resources they employ to overcome these assessments are to use them as motivation to demonstrate their own abilities. So much so, that some participants mentioned personal growth and overcoming these stereotypes as a reason for staying in the education system.

## Limitations and future research

The technique of data collection used, which consisted of conducting interviews in focus groups, is considered particularly suitable for this type of work, as it is intended to explore participants’ knowledge and experience in depth, as well as to create a more stimulating and empathic environment for participation, for participants to open up and to promote active listening (Stewart & Shamdasani, 2014). In addition, in previous studies using individual interviews, students were less motivated to talk and to speak about their personal experiences. However, for future research, an individual interview conducted after the focus groups, with a selection of participants who showed particularly interesting trajectories through the education system, would allow to expand the information on resources, aids and obstacles for these students to stay in post-compulsory studies.

Secondly, it would be of interest to carry out a complementary interview, both at an individual level and through focus groups, with the main agents that have served as support in the educational continuity of the students: family and teachers. Seeing whether the image that students have of how others see them is accurate and triangulating all this information can help students, teachers and families to review their own discourses and the effects that those discourses are having on students’ performance expectations and abilities. These two aspects are the main focus of our future research plans.

## Practical implications

As other authors have stated (Pinya Medina et al., 2017) , data of the nature of those presented in this paper are fundamental for identifying risk and protection factors, above all, favouring the resources and aids that students rely on to stay in the post-compulsory education system, within a prevention and intervention approach. Identifying the personal, academic, affective and social resources that both students and the main support agents put into play, as well as detecting existing problems from the students’ perspective, will make it possible to build the knowledge necessary to design and improve the psychosocial intervention programmes currently being developed in the Areas in Need of Social Transformation (ZNTS).

In this research, we have observed that the attributions of group of students are characterized by their personal agency and individual descriptions of control over their behaviour. Consequently, what can be affirmed from the data obtained is that school intervention should be characterized by the promotion of a positive model of development based on the factors that have contributed to the success shown by resilient adolescents. Specifically, the educational intervention must promote strategies for academic success based on personal effort and internal control, along with programs to develop skills for self-management. Schools must be prepared to encourage attributions which rely on effort and interpretations of failure based on lack of personal work. It is meaningful for educational managers and teachers to become aware of the motivation models built on the sense of learning and the projection of academic success in improving future living conditions. To do this, they must receive support and strategies that make it possible. It is necessary, therefore, to develop and implement intervention programs that are not generic in nature, but are directed at this specific population. In essence, the educational community, at its various levels and responsibilities, must be sensitive to a positive profile of resilience and academic success such as the one shown in this work, in order to respond to the demands and strengths of the educational system in communities at risk of exclusion and, specifically, to counteract school dropout when it comes to post-compulsory education.

## Data Availability

Data cannot be shared openly to protect study participant privacy. Participants are adolescents (minor age). The datasets, instruments and code systems generated during and/or analysed during the current study are available from the corresponding author on reasonable request.
